# Reduced airborne transmission of SARS-CoV-2 BA.1 Omicron virus in Syrian hamsters

**DOI:** 10.1371/journal.ppat.1010970

**Published:** 2022-12-02

**Authors:** Adrianus C. M. Boon, Tamarand L. Darling, Peter J. Halfmann, John Franks, Richard J. Webby, Dan H. Barouch, Julia R. Port, Vincent J. Munster, Michael S. Diamond, Yoshihiro Kawaoka

**Affiliations:** 1 Department of Medicine, Washington University School of Medicine in St. Louis, Missouri, United States of America; 2 Department of Pathology and Immunology Washington University School of Medicine in St. Louis, Missouri, United States of America; 3 Department of Microbiology, Washington University School of Medicine in St. Louis, Missouri, United States of America; 4 Department of Pathobiological Sciences, School of Veterinary Medicine, University of Wisconsin, Madison, Wisconsin, United States of America; 5 Department of Infectious Diseases, St. Jude Children’s Research Hospital, Memphis, Tennessee, United States of America; 6 Center for Virology and Vaccine Research, Beth Israel Deaconess Medical Center, Boston, Massachusetts, United States of America; 7 Ragon Institute of MGH, MIT, and Harvard, Cambridge, Massachusetts, United States of America; 8 Laboratory of Virology, Division of Intramural Research, National Institute of Allergy and Infectious Diseases, National Institutes of Health, Hamilton, Montana, United States of America; 9 The Andrew M. and Jane M. Bursky Center for Human Immunology and Immunotherapy Programs, Washington University School of Medicine. St. Louis, Missouri, United States of America; 10 Department of Virology, Institute of Medical Science, University of Tokyo, Tokyo, Japan; 11 The Research Center for Global Viral Diseases, National Center for Global Health and Medicine Research Institute, Tokyo, Japan; Boston Children’s Hospital, UNITED STATES

Since the start of the COVID-19 pandemic, Severe Acute Respiratory Syndrome Coronavirus 2 (SARS-CoV-2) has caused hundreds of millions of infections worldwide with more than 6.5 million confirmed deaths. SARS-CoV-2 is a respiratory virus that predominantly infects the epithelium of the upper and lower respiratory tract of human and animal hosts. Transmission of respiratory viruses between hosts can occur via direct contact, contact with infected surfaces, or via aerosolized particles [1]. Syrian hamsters are highly susceptible to SARS-CoV-2 infection and one of several animal hosts that have been naturally infected by this virus [2–14]. Transmission of SARS-CoV-2 from pet Syrian hamsters to humans has also been reported [2]. Currently, Syrian hamsters are the only rodent model in which airborne transmission can easily be tested [2,15]. Transmission of SARS-CoV-2 can be established by detection of viral RNA, RNA replication intermediates, viable infectious virus, or seroconversion, with the latter two metrics being the most stringent.

**Fig 1:**
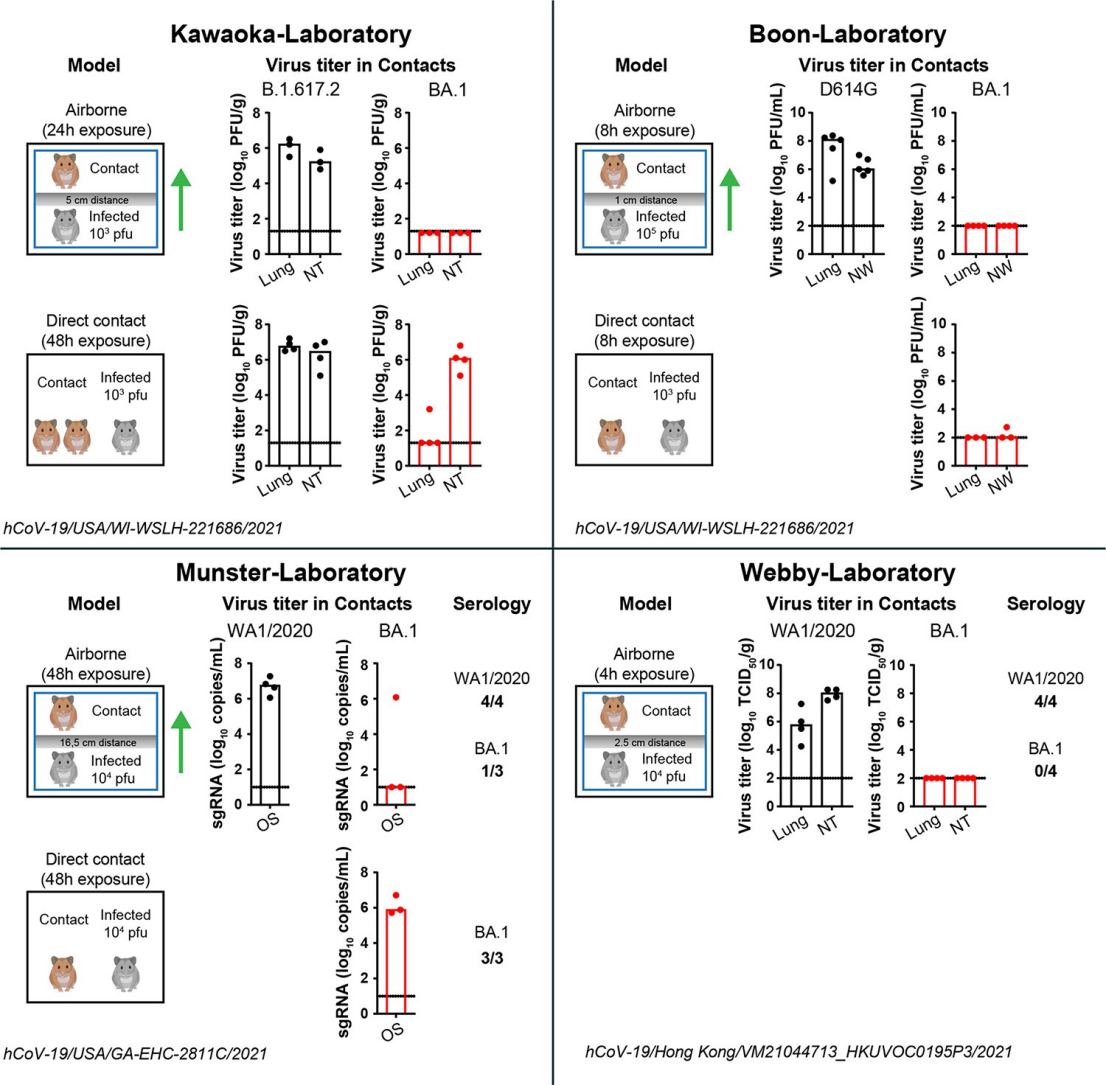
Inefficient airborne transmission of BA.1 variant of SARS-CoV-2 in Syrian hamsters. Experimental design and virus titers in the contact animals exposed to different isolates of the Omicron variant of SARS-CoV-2. **Kawaoka-laboratory**: Five- to six-week-old female Syrian hamsters were inoculated IN with 10^3^ PFUs of the B.1.617.2 (Delta) or BA.1 (Omicron) variant and used as donor animals (grey hamster) in an airborne and direct contact transmission setup. Virus titer in the lungs and NT of the contact animals (brown hamster) was measured by plaque assay 3 dpe. **Boon-laboratory**: Five- to six-week-old male Syrian hamsters were inoculated IN with 10^5^ PFU of a WA1/2020 virus engineered with a D614G mutation (D614G) or the BA.1 variant and used as donor animals in an airborne transmission setup. In a separate experiment, hamsters were inoculated IN with 10^3^ PFU of the BA.1 variant and used as donor animals in a direct contact transmission setup. Virus titer in the lungs and NWs of the contact animals was measured by plaque assay 3 dpe. **Munster-laboratory**: Four- to six-week-old male and female Syrian hamsters were inoculated IN with 10^4^ PFU of WA1/2020 or BA.1 variant and used as donor animals in an airborne transmission setup. Additional hamsters were inoculated IN with 10^4^ PFU of the BA.1 variant and used as donor animals in a direct contact transmission setup. Virus titer in OSs taken from the contact animals was measured by RT-qPCR assay 3 dpe, and seroconversion was measured 14 dpe by spike antigen ELISA. **Webby-laboratory**: Eight- to ten-week-old male Syrian hamsters were inoculated IN with 10^4^ PFU of the WA1/2020 or BA.1 variant and used as donor animals in an airborne transmission setup. Virus titer in the lungs and NT of the contact animals was measured by infectious virus assay 4 dpe, and seroconversion was measured 21 dpe by virus neutralization assay. Each symbol is an individual animal and the bars represent the median value. Dotted line indicates the limit of detection for each respective assay. Green arrow indicates directional airflow. This figure was created with a BioRender template (hamster image). dpe, days postexposure; IN, intranasally; NT, nasal turbinate; NW, nasal wash; OS, oral swab; PFU, plaque-forming unit; RT-qPCR, reverse transcription quantitave PCR.

Since January 2021, the National Institutes of Health SARS-CoV-2 Assessment of Variant Evolution (SAVE) initiative has evaluated the transmission potential of SARS-CoV-2 variants in Syrian hamsters [16]. Several SAVE-affiliated laboratories, using variable exposure times (4 to 48 hours), inoculum amounts (10^3^ to 10^5^ infectious units), and experimental designs, observed airborne transmission of SARS-CoV-2 (strain Wuhan-1 or WA1/20202) to nearly 100% of the contact animals. Similar results were obtained with the B.1.1.7 (Alpha), B.1.351 (Beta), and B.1.617 (Delta) variants of SARS-CoV-2 [15,17,18]. Airborne transmission was associated with high levels of viral RNA and infectious virus in respiratory tissues of the contact animal, and when tested, seroconversion (**Fig 1**). Certain amino acid mutations (D614G and N501Y) found in the receptor-binding domain of the spike protein of many SARS-CoV-2 variants improved airborne transmission of SARS-CoV-2 in hamsters [19–22]. Late in 2021, the antigenically distinct BA.1 (Omicron) variant emerged. BA.1 viruses were shown to be less pathogenic than prior variants in mice and Syrian hamsters [23,24]. Four SAVE-affiliated laboratories also evaluated BA.1 transmission between hamsters (**Fig 1**). In direct contact transmission models evaluated in three different laboratories, infectious BA.1 virus was found in 8/10 (80%) of the contact animals. Seroconversion was measured by one laboratory and detected in all three contacts. In contrast, in airborne transmission models, where infected and contact animals are physically separated, infectious BA.1 virus was detected only in 1/13 (7.5%) contact animals. Seroconversion, evaluated in two different laboratories, was detected in the one SARS-CoV-2 RNA replication intermediate positive contact animal. These data suggest that BA.1 airborne transmission is substantially less efficient in hamsters than prior variants. Similar results were obtained for the BA.2.12.2 and BA.5 Omicron variant of SARS-CoV-2. The observations from the SAVE network agree with a recent report [24] evaluating the transmission of BA.1 and BA.2 in hamsters, but contrast with those of Yuan and colleagues [25] who reported, using viral RNA detection methods, efficient airborne transmission of the BA.1 (Omicron) variant of SARS-CoV-2 between hamsters. These results highlight how the metrics used to establish airborne transmission can impact study conclusions, which could affect animal model utilization and even public health messaging.

Notwithstanding these points, more work is required to understand how well hamsters model SARS-CoV-2 transmission phenotypes in humans, especially for emerging variants. The observation that the BA.1 variant of SARS-CoV-2 does not transmit efficiently via the air in Syrian hamsters appears to contrast with human epidemiological data. The reason for this difference in BA.1 transmission between humans and hamsters is not clear. One possible explanation is the sequence difference in angiotensin-converting enzyme 2 (ACE2) receptor [26], which could impact replication and transmission efficiency of BA.1 in hamsters. The reported attenuation of BA.1 in Syrian hamsters supports this observation [23,24]. However, BA.1 was also attenuated in human ACE2-transgenic hamsters [23], implying that genetic differences in non-spike proteins, which can modulate innate and antiviral immunity, also contribute to the loss of airborne transmission of BA.1 in this model. It has also been reported that the Omicron variant is less dependent on the protease TMPRSS2 for spike protein cleavage and more dependent on cathepsins than the other variants [27–30], implying that the Omicron variant enters cells through a different route. This may have differential effects in humans and hamsters and reduce airborne transmission efficiency of Omicron variants between hamsters. Finally, it is possible that the changes in the spike protein of Omicron affect and reduce stability of the virus in the infected hosts. Differences in pH, salinity, or mucosal barrier between humans and hamsters before or after SARS-CoV-2 infection could contribute to differences in airborne transmission of the BA.1 Omicron variant between humans and hamsters.

To summarize, the combined analysis from multiple groups using different isolates of the BA.1 Omicron variant demonstrate that BA.1 has reduced airborne transmission efficiency in Syrian hamsters compared to prior variants of SARS-CoV-2.
